# Intestinal β-carotene bioconversion in humans is determined by a new single-sample, plasma isotope ratio method and compared with traditional and modified area-under-the-curve methods^☆^

**DOI:** 10.1016/j.abb.2018.06.015

**Published:** 2018-09-01

**Authors:** Jennifer Lynn Ford, Michael H. Green, Joanne Balmer Green, Anthony Oxley, Georg Lietz

**Affiliations:** a Department of Nutritional Sciences, The Pennsylvania State University, University Park, PA 16802, United States; b Human Nutrition Research Centre, Newcastle University, Newcastle upon Tyne, NE2 4HH, UK

**Keywords:** β-Carotene, Carotenoid bioconversion, Carotenoid bioefficacy, Area under the curve, Vitamin A

## Abstract

The vitamin A value (bioefficacy) of provitamin A carotenoids is determined by absorption of the carotenoid (bioavailability) and its subsequent conversion to retinol (bioconversion). Here we show that intestinal bioconversion of β-carotene can be estimated based on analysis of a single plasma sample collected 6 h after subjects ingest a test dose of stable isotope-labeled β-carotene from the ratio of retinyl esters to retinyl esters plus β-carotene. Plasma isotope ratio predictions of bioconversion ranged from 50 to– 93% (mean 76%) for 45 healthy young adults with low vitamin A stores. Results were the same as predictions made by a traditional area-under-the-curve method calculated from 0 to– 8 h or a modified area-under-the-curve method calculated from 0 to– 12 h. The modified method may provide better estimates of bioconversion between 8 and 24 h after ingestion of a carotenoid dose when stable isotopes cannot be used due to cost or logistics. Furthermore, because the plasma isotope ratio method requires only one blood sample and no isolation of triglyceride-rich lipoproteins, its use will facilitate estimation of provitamin A carotenoid bioconversion in human subjects and especially children, in whom repeated blood sampling is not feasible.

## 1 Introduction

Provitamin A carotenoids, especially β-carotene (βC), are a significant source of vitamin A in the diet of many individuals worldwide, accounting for about 70% of vitamin A intake in many lower income areas [1,2]. Since the vitamin A value of provitamin A from different food sources is affected by many factors [3], researchers have sought to develop simple and accurate methods for estimating bioavailability (the extent of carotenoid absorption), bioconversion (the extent of conversion of the absorbed carotenoid to retinol), and bioefficacy (the product of those two factors, or the proportion of ingested carotenoid that is absorbed and converted to retinol) [4,5]; for reviews, see Refs. [1,2,4,6].

In the current work, given that genetic variations in β-carotene 15,15′-oxygenase (BCO1) have been shown to contribute to the large inter-individual variation observed in βC conversion efficiency [7,8], we compared methods for estimating βC bioconversion. Previously, βC bioconversion has been estimated based on areas under plasma concentration curves (AUC) for labeled βC and retinyl esters (RE) after subjects ingest a dose of labeled βC [[9], [10], [11]]; estimates ranged from 35 to 88% [9,12,13]. Such AUC methods for determining bioconversion require the collection of multiple blood samples over time and frequently include the isolation of plasma triglyceride-rich lipoproteins; each of these considerations can limit the usefulness of the AUC method, especially in children in whom repeated blood sampling is difficult.

Recently [14], we validated the use of a plasma retinol isotope ratio method for estimating carotenoid relative bioefficacy based on analysis of a single blood sample obtained after previously-studied subjects [15,16] consumed a dose of labeled βC and a reference dose of labeled retinyl acetate. Here we extend that method to estimate intestinal βC bioconversion, showing that one obtains comparable results from a plasma isotope ratio (IR) and two AUC methods. We also present a correction method to improve predictions of bioconversion estimated by the traditional AUC method. Use of the single-sample, whole plasma method to estimate intestinal βC bioconversion will allow this parameter to be more easily determined in human subjects, including children.

## 2 Methods

### 2.1 Subjects and data collection

We used data from 45 individuals previously described by Oxley et al. [15]; see also Green et al. [16]. In brief, after an overnight fast, healthy young men and women received doses of [^13^C_10_]retinyl acetate (1 mg) and [^13^C_10_]βC (2 mg) in sunflower oil via an oral syringe. They then consumed a breakfast meal that contained 46 g fat, 64 g carbohydrate and 10 g protein (717 kcal). Four h later, participants had a lunch meal that contained 37 g fat, 63 g carbohydrate and 18 g protein (655 kcal); a dinner meal consumed 8 h after dose administration provided 20 g fat, 43 g carbohydrate and 18 g protein (440 kcal); water was the only beverage offered on d 1. After the first day of the study, subjects were asked not to deviate from their habitual diets. All procedures involving human subjects were approved by the National Research Ethics Service, North East–Sunderland Committee (REC 09/H0904/20) and the study was registered with the UK Clinical Research Network (UKCRN: 7413).

Ten blood samples were collected from each subject from 2 h to 14 d after dose administration; for the current work, we used data for samples obtained from 2 to 24 h (2, 4, 6, 8, 10, 12 and 24 h; n = 7). Plasma was analyzed by LC-MS/MS for [^13^C_10_]RE and [^13^C_10_]retinol derived from the retinyl acetate dose and for [^13^C_10_]βC, [^13^C_5_]RE and [^13^C_5_]retinol derived from the βC dose [15]. For the current analysis, we examined data for [^13^C_10_]βC and for the [^13^C_5_]RE derived from [^13^C_10_]βC.

### 2.2 Estimation of intestinal bioconversion of β-carotene

For each subject, plasma concentration of labeled RE (μmol/L) was converted to molar βC equivalents (RE_βCe_). That is, because 1 molecule of all-*trans*-βC could maximally yield 2 molecules of retinol, we calculated [^13^C_5_]RE_βCe_ as [^13^C_5_]RE/2. Plasma concentrations (μmol/L) of labeled βC and labeled RE_βCe_ were plotted versus time after dose administration. Then, intestinal bioconversion of absorbed βC was calculated at 2, 4, 6, 8, 10, 12 and 24 h after dosing as a plasma isotope ratio (IR method): {[^13^C_5_]RE_βCe_/([^13^C_5_]RE_βCe_ + [^13^C_10_]βC)} × 100.

In addition to the IR method, intestinal bioconversion of absorbed βC was calculated using a traditional AUC method as {AUC for [^13^C_5_]RE_βCe_/(AUC for [^13^C_5_]RE_βCe_ + AUC for [^13^C_10_]βC)} × 100, where areas under the plasma isotope response curves [(μmol/L) × h] were integrated from 0 to 2, 4, 6, 8, 10, 12 or 24 h. Also, we developed an adjusted AUC method (AUC_adj_) that corrects for βC in very low density lipoproteins (VLDL) because at later times, labeled βC in plasma will be transported not only in chylomicrons but also in VLDL secreted by the liver. The rationale for the AUC_adj_ method is that incorporation of βC and RE into nascent chylomicrons is linked [17,18] and if we assume that the ratio of βC to RE in chylomicrons remains relatively constant, we can use labeled RE as a proxy for chylomicron βC. For the AUC_adj_ method, we calculated chylomicron βC concentrations as [^13^C_10_]βC at 6 h × {[^13^C_5_]RE at 8, 10, 12 or 24 h/[^13^C_5_]RE at 6 h}, assuming that at 6 h, labeled βC was primarily in chylomicrons and any contribution from VLDL would be minimal (i.e., VLDL βC at 6 h = 0 and total βC concentration at 6 h = chylomicron βC concentration at 6 h). Specifically, we calculated ratios for [^13^C_5_]RE concentration at 8, 10, 12 and 24 h versus [^13^C_5_]RE at 6 h for each subject (see Results); the ratios at these times were then multiplied by individual subject values for [^13^C_10_]βC concentration at 6 h to derive estimated chylomicron βC concentrations from 8 to 24 h.

Then, estimated βC chylomicron concentrations were used to calculate intestinal bioconversion as {AUC for [^13^C_5_]RE_βCe_/(AUC for [^13^C_5_]RE_βCe_ + AUC for chylomicron [^13^C_10_]βC)} × 100.

Estimates of intestinal bioconversion predicted by the IR method and the two AUC methods were compared graphically and statistically as described later.

### 2.3 Data manipulations and statistics

Data are presented as means ± SD (range) or as means ± SEM. Areas under plasma isotope response curves were integrated by application of the trapezoidal rule using GraphPad Prism v. 7.0 (GraphPad Software Inc.). Figures and data simulations were also done using GraphPad Prism v. 7.0. Predictions of intestinal bioconversion by the IR and AUC methods were compared using mixed models ANOVA (with ‘method’, ‘time’ and ‘method × time’ as fixed effects, and ‘subject’, ‘subject × method’ and ‘subject × time’ as random effects) and two post-hoc tests: the Tukey honest significant difference test to determine within-subject differences and the two one-sided tests approach to test equivalence using a threshold of 3 bioconversion percentage units (JMP Pro v. 13; SAS Institute Inc.) for the 6 h IR versus 8 h AUC comparison. P < 0.05 was considered significant. Least squares regression analysis was performed using GraphPad Prism v. 7.0.

## 3 Results and discussion

### 3.1 Subject characteristics

Among the 45 healthy subjects, 25 were female and 20 were male; the mean age was 24 ± 4 y and body mass index was 22 ± 2.2 kg/m^2^; plasma retinol concentrations were within the normal range (1.51 ± 0.28 μmol/L). Vitamin A total body stores, estimated for 30 of the subjects studied previously using model-based compartmental analysis [16], averaged 123 ± 71 μmol.

### 3.2 Plasma tracer kinetics

**Fig. 1:**
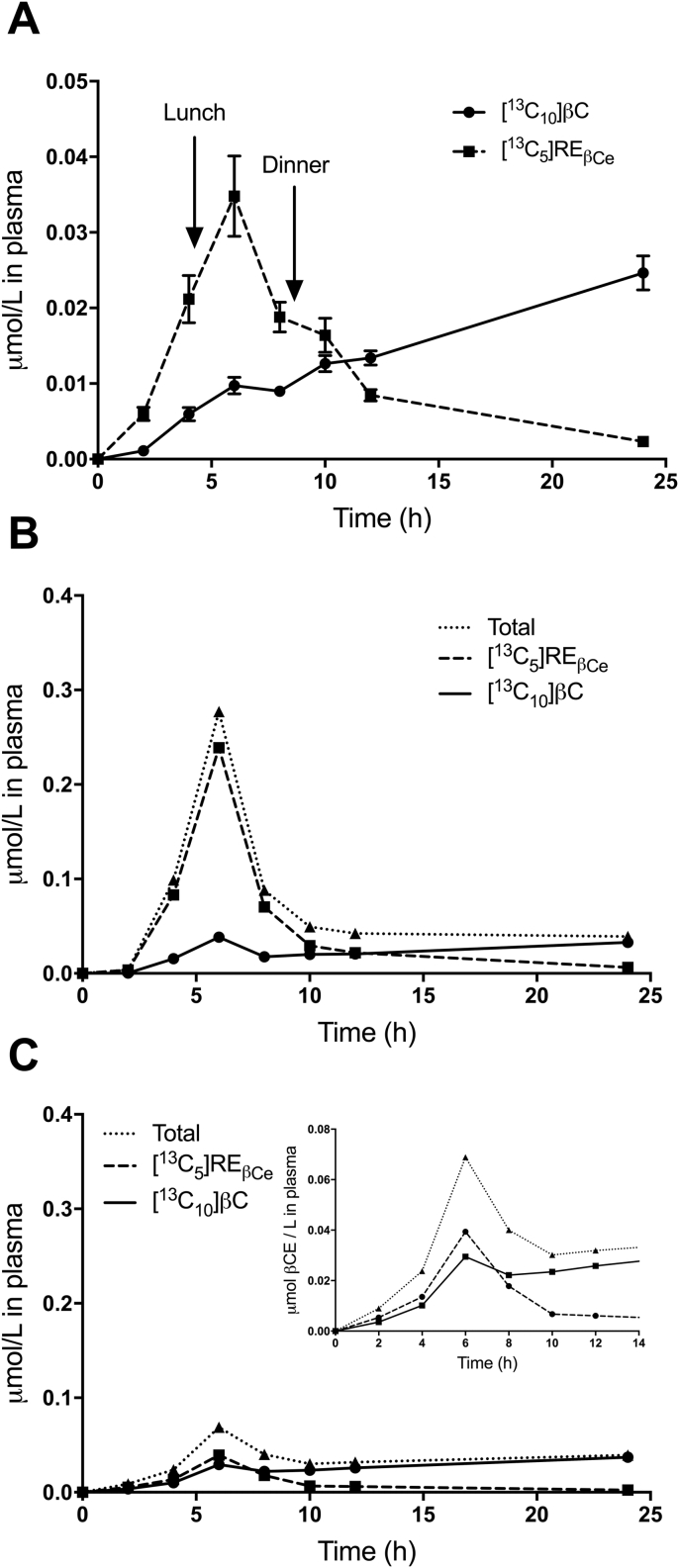
Observed data for concentration of tracers in plasma versus time. Shown are group mean μmol/L ± SEM (n = 45) for [^13^C_10_]βC (circles connected by a solid line) and for [^13^C_5_]RE_βCe_ (squares connected by a dashed line) (panel A). Arrows indicate when lunch and dinner were provided. Also shown are μmol/L versus time for [^13^C_10_]βC (circles connected by a solid line), for [^13^C_5_]RE_βCe_ derived from the βC dose (squares connected by a dashed line), and for the sum of these tracers (reflecting total absorbed [^13^C_10_]βC; triangles and dotted lines) for subjects with high (85%; panel B) and low intestinal bioconversion (55%; panel C). Panel C inset shows tracer concentrations to 14 h for the subject with low bioconversion. Subjects whose data are illustrated in panels B and C were selected based on bioconversion calculated by AUC using data from 0 to -8 h. AUC, area under the curve; βC, β-carotene; RE_βCe_, retinyl esters as molar β-carotene equivalents.

**Fig. 2:**
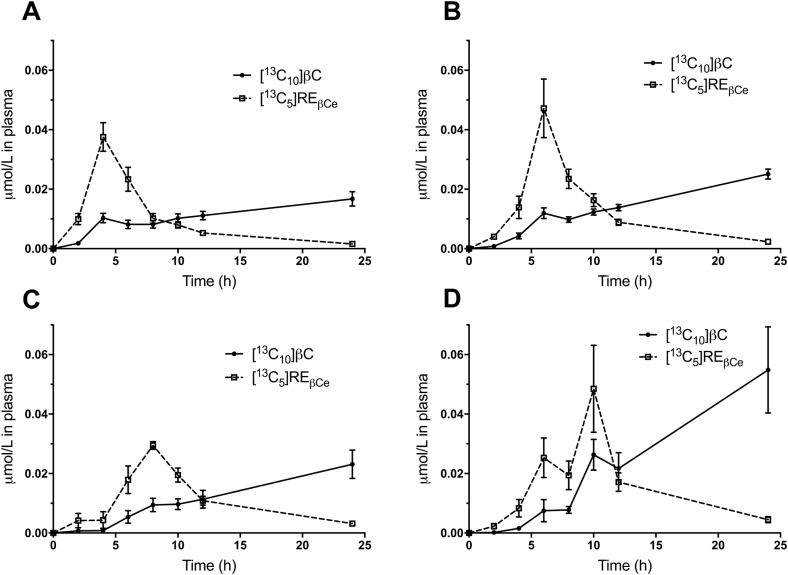
Observed data for plasma tracer concentrations versus time. Shown are mean μmol/L ± SEM for [^13^C_10_]βC (circles connected by a solid line) and for [^13^C_5_]RE_βCe_ (squares connected by a dashed line) for subjects with the highest RE concentrations at 4 (n = 16), 6 (n = 22), 8 (n = 3) or 10 (n = 4) h (panels A–D, respectively). βC, β-carotene; RE_βCe_, retinyl esters as molar β-carotene equivalents.

Mean plasma concentrations versus time for [^13^C_10_]βC and for [^13^C_5_]RE_βCe_ derived from the βC dose are shown in Fig. 1A. The data indicate that labeled βC increased from 2 to 6 h after dose ingestion, decreased from 6 to 8 h, and then further increased after 8 h; in contrast, labeled RE_βCe_ peaked at 6 h and then decreased with time. Similar plots, as well as data for the sum of the two tracers (i.e., [^13^C_10_]βC plus [^13^C_5_]RE_βCe_, with retinyl esters expressed as molar β-carotene equivalents), are shown in Fig. 1B for a subject with high intestinal bioconversion (85%) based on the 8 h AUC method (see next section) and in Fig. 1C for an individual with a lower bioconversion (55%). Note that the curves for the sum of the two tracers reflect absorption of labeled βC. For the individual shown in Fig. 1B, labeled βC followed a time course that was similar to the pattern for the group mean data (Fig. 1A); in contrast, the peak at 6 h for labeled RE_βCe_ was higher than the group mean (0.24 μmol/L versus 0.035 μmol/L) and then RE_βCe_ decreased dramatically with time. For the subject shown in Fig. 1C, the labeled βC response was similar to the group mean response and labeled RE_βCe_ peaked at 6 h (0.039 μmol/L). However, the overall RE_βCe_ response for this subject was much lower than for the individual with higher intestinal bioconversion (Fig. 1B) and for the group mean data (Fig. 1A). For the subject shown in Fig. 1B, total plasma concentration of label (βC plus RE_βCe_) peaked at 0.28 μmol/L, compared with 0.069 μmol/L for the subject with the lower intestinal bioconversion (Fig. 1C), presumably reflecting a higher βC absorption (i.e., bioavailability). As shown in Fig. 2, for the vast majority of subjects (38 of the 45), the maximum concentration of plasma labeled RE_βCe_ was observed at 4 h (n = 16) or 6 h (n = 22) post-dosing; for 3 subjects, the maximum was seen at 8 h, and for 4 subjects, it was at 10 h.

Although in most subjects, plasma RE_βCe_ decreased continuously after the early peak, in a some subjects (n = 10), there was a second peak in RE_βCe_ at 10 h; for 3 of these 10 subjects, the maximum concentration of RE_βCe_ in plasma was observed at the second (10 h) peak (Fig. 2). The initial, predominant peak (at 6 or 8 h) in a given subject is likely the response to the breakfast and lunch meals (consumed after dose administration and after collection of the 4 h blood sample, respectively), whereas when a second peak was observed at 10 h, it is likely an additional response to the dinner meal (consumed after the 8 h sample was collected).

### 3.3 Intestinal β-carotene bioconversion

A summary of the results for intestinal βC bioconversion predicted by IR and AUC is presented in Table 1. Note that results for IR versus AUC are the same at 2 h because only one sample was collected by this time and thus the same data were used for both calculations. Since the plasma concentration of RE_βCe_ was highest relative to βC at 2 h after dosing, the data indicate that bioconversion was most efficient in these subjects at this time. In fact, for all volunteers, [^13^C_5_]RE was detected in the 2 h plasma sample, whereas [^13^C_10_]βC was not detected in plasma for 10 of the 45 subjects at 2 h and for one even at 4 h. By 6 h, both labeled βC and its RE metabolite were detected in plasma of all subjects.

**Table 1:** Intestinal βC bioconversion estimated by an IR and two AUC methods^A^.

Time (h)	IR (%)	AUC (%)	${\mathrm{AU}C_{\mathrm{adj}}}^{B}$ (%)
2	87.2 ± 10.2 (60.0–100.0)	87.2 ± 10.2 (60.0–100.0)	87.2 ± 10.2 (60.0–100.0)
4	79.0 ± 11.0^abc^ (50.3–100.0)	81.7 ± 8.9^ab^ (58.4–100.0)	81.7 ± 8.9^a^ (58.4–100.0)
6	76.0 ± 11.1^cd^ (49.9–92.6)	78.8 ± 9.3^abc^ (57.5–91.3)	78.8 ± 9.3^abc^ (57.5–91.3)
8	63.4 ± 15.6^f^ (34.6–84.2)	76.2 ± 9.9^c^ (55.3–88.8)	78.0 ± 9.7^bc^ (57.4–92.0)
10	52.4 ± 16.1^g^ (21.9–80.4)	72.2 ± 10.8^d^ (50.7–86.0)	77.7 ± 9.8^c^ (57.4–92.4)
12	38.5 ± 14.0^i^ (10.0–62.3)	67.8 ± 11.7^e^ (45.8–83.7)	77.5 ± 9.9^c^ (57.3–92.5)
24	9.5 ± 5.3^j^ (0.93–28.9)	45.8 ± 13.1^h^ (21.9–70.0)	77.2 ± 10.1^c^ (57.3–92.6)

^A^Values are means ± SD (range) for percent intestinal β-carotene bioconversion predicted using a plasma isotope ratio method compared to two area-under-the-curve methods (n = 45 at each time). ^B^AUC for intestinal βC bioconversion calculated using the estimated chylomicron βC concentrations that were corrected for very low density lipoprotein βC; see text for details. Means with different superscript letters are significantly different (P < 0.05) based on the significant ‘method × time’ interaction determined by mixed models ANOVA. Note that predictions at 2 h were identical for the 3 methods and thus were excluded from the mixed model analysis. AUC, area under the curve; AUC_adj_, adjusted AUC; βC, β-carotene; IR, isotope ratio.

**Fig. 3:**
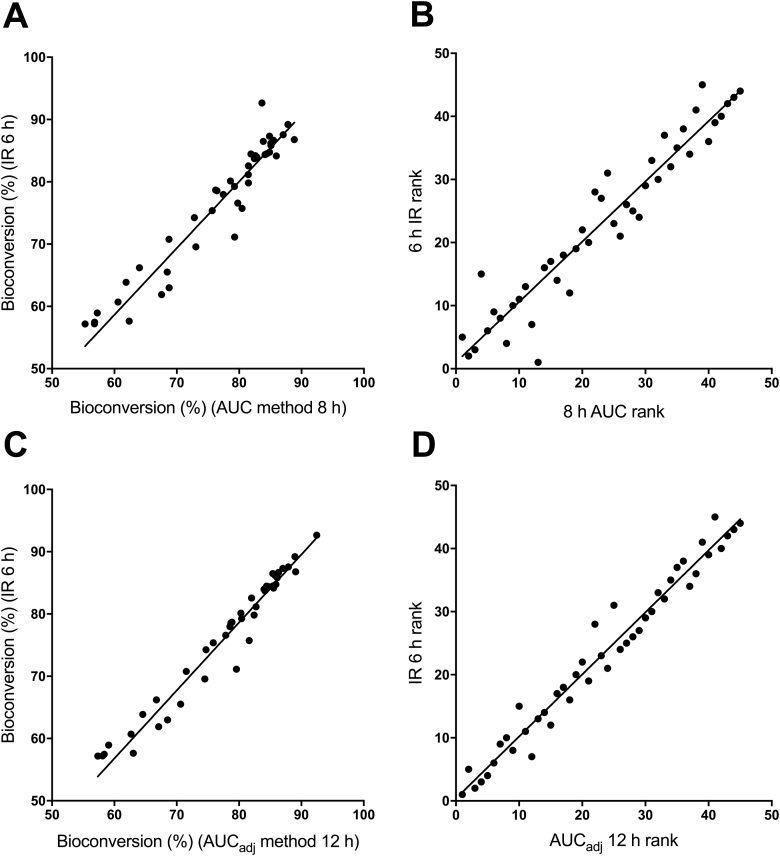
Bioconversion estimated by an IR method and two AUC methods. Intestinal βC bioconversion calculated by the IR at 6 h versus AUC at 8 h is shown in panel A; IR at 6 h versus AUC_adj_ at 12 h is presented in panel C; n = 45. See text for methods of calculation. Least squares regression lines were y = 1.0x – 3.2 (R^2^ = 0.91; P < 0.0001) (panel A) and y = 1.0x – 0.62 (R^2^ = 0.95; P < 0.0001) (panel C). Rank-order correlations are shown in panel B for AUC (R = 0.96, P < 0.0001) and in panel D for AUC_adj_ (R = 0.98, P < 0.0001). AUC, area under the curve; AUC_adj_, adjusted AUC; βC, β-carotene; IR, isotope ratio.

In the case of the IR method (Table 1), mean predictions were highest at 2 h (87%) and then decreased to 63% at 8 h; the two subsequent values were substantially lower at each time, reaching 10% at 24 h. In contrast, for the traditional AUC method (column 3), the initially high value at 2 h gradually decreased to 68% at 12 h; by 24 h, the prediction was substantially lower (46%). When comparing the IR and AUC methods, mean predictions were not significantly different at 4 and 6 h. At times from 8 h on, IR predictions of bioconversion were significantly lower than those calculated by AUC. For the AUC method, we used 8 h as a basis for comparison to the IR since past studies were carried out only until 8 [9] or 9 h after dosing [10]. In addition, up to 8 h, the influence of incoming VLDL βC on area calculations for βC and thus on bioconversion is less than at later times (see next paragraph). Compared to mean predictions by the AUC method at 8 h (76%), mean predictions by the IR at 4 h (79%) and 6 h (76%) were not significantly different from each other or from the AUC result at 8 h. However, values predicted by the IR at 6 h were closer to values calculated by the AUC at 8 h and in fact, based on the two one-sided tests, the means were practically equivalent (to within our threshold of 3 bioconversion units; P = 0.0054); the mean ratio of IR at 6 h/AUC at 8 h was 1.0 (range, 0.80–1.1). As shown in Fig. 3A, individual subject predictions by the IR at 6 h were strongly and significantly correlated with values calculated using the AUC method at 8 h (R^2^ = 0.91; P < 0.0001). When predictions were ranked (Fig. 3B), we found a strong and significant rank-order correlation (R = 0.96, P < 0.0001). Since, in the field, one would not know the time at which plasma RE peaks in a given subject, we compared predictions by the IR at 6 h and the AUC at 8 h for subjects sorted by the time that a first peak in RE was observed (4, 6, 8 or 10 h) (Fig. 2). Overall, for subjects in whom the peak was observed at 4, 6 or 8 h, the mean ratio of 6 h IR/8 h AUC was within 3 bioconversion percentage units. The first RE peak was observed at 10 h for only 1 subject (i.e., for the other 3 subjects in whom a 10 h peak was observed, it was a second peak); in that subject, the ratio of 6 h IR/8 h AUC was 1.1. Based on these results, we suggest that 6 h may be an optimal time to use the IR method to predict intestinal bioconversion.

The decreases we observed in intestinal βC bioconversion over time are compatible with current understanding of the formation and metabolism of triglyceride-rich lipoproteins. Specifically, the initial decrease (2 to 6 h) is likely explained by a delay in the incorporation of βC versus βC-derived retinyl esters into chylomicrons [9]; by 8 h, the continuing decrease is likely due to appearance of labeled βC in VLDL [19]. Presumably, predictions by the plasma IR method after 6 h decreased at a faster rate compared with AUC because VLDL appearance in plasma will have a greater effect on the ratio at any single time compared with its impact on the cumulative area used to calculate AUC. In addition, note that the IR method at 6 h predicts bioconversion of βC to RE during absorption of components of the breakfast meal and the early post-prandial response to the lunch consumed after collection of the 4 h blood sample.

**Fig. 4:**
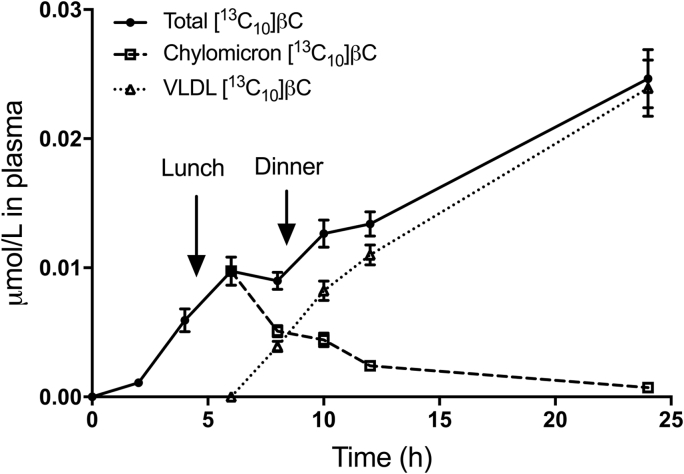
Observed and estimated data for concentration of tracers in plasma versus time. Shown are group mean ± SEM (n = 45) observed μmol/L for [^13^C_10_]βC (circles and solid line) and simulated data for βC in chylomicrons (squares and dashed line) versus VLDL (triangles and dotted line). Arrows indicate when lunch and dinner were provided. βC, β-carotene; VLDL, very low density lipoproteins.

Since it is known that, at early times after administration of labeled carotenoid, βC in plasma is found exclusively in chylomicrons but at later times, it is also present in VLDL, we estimated the likely time course for the labels in chylomicrons versus VLDL as a fraction of the total βC concentration. As shown in Fig. 4, simulations indicate that mean βC in chylomicrons peaked by 6 h after dosing and that by 8 h, βC was also present in VLDL; by 10 h, βC in VLDL slightly exceeded βC in chylomicrons. At 12 h, most of the labeled βC appeared to be transported in VLDL and even more so by 24 h.

As stated earlier, we also calculated intestinal βC bioconversion using estimated chylomicron βC concentrations for the adjusted AUC method. For this method, we determined that mean (±SD) [^13^C_5_]RE_βCe_ concentrations at 8, 10, 12 and 24 h relative to those at 6 h were 66 ± 48% (8 h), 67 ± 85% (10 h), 34 ± 34% (12 h) and 6.7 ± 8% (24 h); then [^13^C_10_]βC concentrations at 6 h for each subject were multiplied by their fractional change in RE_βCe_ at these times to estimate βC concentrations in chylomicrons at 8, 10, 12 and 24 h. Predictions of bioconversion calculated using the AUC_adj_ method stabilized and were relatively constant from 8 to 24 h (Table 1). In addition, as also shown in the table, if total βC is corrected for βC in VLDL, mean AUC predictions at 8 h and IR predictions at 6 h agree with AUC_adj_ predictions from 8 to 24 h. When we compared values predicted by the IR at 6 h to values calculated by the AUC_adj_ at 12 h, the mean ratio of IR at 6 h/AUC_adj_ at 12 h was 0.98 (range, 0.81–1.0). As shown in Fig. 3C, individual subject predictions by the IR at 6 h were strongly and significantly correlated with values calculated using the AUC_adj_ method at 12 h (R^2^ = 0.95; P < 0.0001). When ranked (Fig. 3D), predictions were strongly and significantly correlated (R = 0.98, P < 0.0001). These results further support the use of the IR at 6 h to predict intestinal bioconversion.

Previous researchers have reported that βC bioconversion ranged from 35 to 88% [9,12,13]; our current 6 h IR predictions range from 50 to– 93% (mean, 76%). This relatively high bioconversion may relate to the low vitamin A total body stores in these subjects (123 μmol for 30 of the 45 individuals studied by Green et al. [16]). In addition, as previously reported [20,21], the efficiency of βC absorption and its conversion to retinol (i.e., bioefficacy) is highly variable among individuals. For the current subjects, the coefficient of variation (CV) for intestinal βC bioconversion predicted by the 6 h IR was 14%. As found in the earlier study by Green et al. [16], in which relative bioefficacy was estimated for 30 of these 45 subjects, the CV for that parameter was 44%. Taken together, these results suggest that the large inter-individual variation in bioefficacy is more likely due to high variability in the absorption of βC (i.e., bioavailability) rather than in its conversion efficiency.

### 3.4 Concluding comments

In view of current results for the plasma isotope ratio method for estimating intestinal βC bioconversion, it would be worthwhile to apply the IR and the adjusted AUC method retrospectively to previously-collected data from studies that have used AUC methods and to compare results obtained by the three methods. One limitation of both the IR and AUC methods to estimate intestinal bioconversion of carotenoids is that there is no “reference method” that can be used to validate the results. We suggest that, in subsequent work, researchers could apply the tools of model-based compartmental analysis of theoretical data to generate “known values” for bioavailability and bioconversion that could be compared with IR and AUC calculations done on the same data. We [14] recently used such a strategy to validate a plasma retinol isotope ratio method for estimating βC bioefficacy at 14 or more days after dosing with labeled βC and retinyl acetate (reference dose).

In conclusion, we have shown that the ratio of plasma concentration in retinyl esters to that in retinyl esters plus β-carotene in a single plasma sample collected 6 h after subjects ingest a test dose of labeled β-carotene provides an estimate of intestinal bioconversion of β-carotene that is comparable to area-under-the-curve estimates which require analysis of multiple blood samples. The ability to estimate bioconversion based on analysis of a single sample of whole plasma will make it feasible to measure intestinal β-carotene bioconversion in human subjects, and especially in children.

## Author disclosure statement

The authors declare no competing financial interests.

## Abbreviations used

AUC, area under the curve; AUC_adj_, adjusted AUC; βC, β-carotene; IR, isotope ratio; RE, retinyl esters; RE_βCe_, retinyl esters as molar β-carotene equivalents; VLDL, very low density lipoproteins.
